# The Effects of Integrative In-Patient Treatment on Patients' Quality of Life: A Meta-Analysis

**DOI:** 10.1155/2013/416510

**Published:** 2013-01-28

**Authors:** Thomas Ostermann, Jost Langhorst, Andre-Michael Beer

**Affiliations:** ^1^Center of Integrative Medicine, Witten/Herdecke University, Gerhard-Kienle-Weg 4, 58313 Herdecke, Germany; ^2^Department of Internal and Integrative Medicine, Kliniken Essen-Mitte, University of Duisburg-Essen, 45276 Essen, Germany; ^3^Department of True Naturopathy, Blankenstein Hospital, Im Vogelsang 5-11, 45527 Hattingen, Germany

## Abstract

*Background*. In the last decades, several hospitals have adopted this concept of integrative medicine for the treatment of chronic and acute states of illnesses in in-patient treatment. The aim of this paper was to summarize the current evidence for a possible effectiveness of integrative on-patient treatment in patients' quality of life by means of a meta-analysis. *Material and Methods*. The databases MEDLINE, EMBASE, AMED, PsycInfo, PsycLit CCMED, and CAMbase were screened to find articles. We also screened publisher databases to find relevant information. Articles were included if patients were treated in a hospital. To guarantee comparability SF-36 was the predefined outcome measure for patients' quality of life. Data of pre/posteffects on the mental and physical scores of the SF-36 were extracted and effect sizes were calculated and entered into a random effect meta-analysis. *Results*. Eight articles published between 2003 and 2010 were included in the final meta-analysis. Random effect meta-analysis of the eight studies revealed an overall effect size of 0.37 (95% CI: [0.28; 0.45]) in the physical score and 0.38 (95% CI: [0.30; 0.45]) in the mental score of the SF-36. *I* ^2^ statistics indicate a high heterogeneity in the effects in both the physical and mental scores of the SF-36 (*I* ^2^ = 91.8%, *P* < 0.001, resp.; *I* ^2^ = 86.7%, *P* < 0.001). *Discussion*. This meta-analysis might help to rediscover the importance of integrative in-patient treatment for patients, physicians, and stakeholders.

## 1. Introduction

Integrative medicine according to the definition of the consortium of the Academic Health Centers for integrative medicine is “the practice of medicine that reaffirms the importance of the relationship between practitioner and patient, focuses on the whole person, is informed by evidence, and makes use of all appropriate therapeutic approaches, healthcare professionals, and disciplines to achieve optimal health and healing” [1]. It therefore may combine the treatment of conventional medicine and complementary alternative medicine (CAM) and assists the patient's own capacities to recover from illness. 

In the last decades, several hospitals have adopted this concept of integrative medicine for the treatment of chronic and acute states of illnesses in in-patient treatment [2, 3]. This includes hospitals with a special focus on mind body therapies, naturopathy, anthroposophical medicine, homeopathy or traditional Chinese medicine. From those institutions, a variety of high-quality clinical studies in special therapies like acupuncture [4], leeches therapy [5], fasting [6], or cupping [7] have been performed and published which demonstrate the power of single components of integrative in-patient treatment. Moreover large studies have also investigated safety aspects of these approaches [8]. To provide additional evidence for the whole system in real world treatment, concept evaluations of the approach of integrative medicine for in- and out-patient treatment have been proposed [9].

Already in the very early years of these institutions such whole systems evaluations, that is, with the focus on comparative health economic analysis, demonstrated the therapeutic potential of these approaches [10]. Nowadays such evaluations have regained the interest of stakeholders of the health care system such as health insurances or governmental authorities mainly to develop special diseases management pathways or to create specific diagnose related groups and additional payments [11, 12]. In particular scientific interest was focused on the sustainability of integrative treatment outcomes after in-patient treatment. Studies in this field so far have shown high patient satisfaction, and reduced out-patient expenses and doctor's visits [13]. 

In the appraisal of patient's benefits several measures like patient' mood, depression, or pain perception were applied to demonstrate the effects of integrative in-patient treatment. However health related quality of life very early became the main and most important outcome parameter and denotes the least common denominator of such evaluations [14].

Up to now, published data is widespread and no systematic review so far has collected the results of the studies to get a broader picture of the effects of integrative in-patient treatment. The aim of this paper was to summarize the current evidence for a possible effectiveness of integrative in-patient treatment on patients' quality of life by means of a meta-analysis.

## 2. Material and Methods

### 2.1. Search Strategy

The following databases were used to find articles: MEDLINE, EMBASE, AMED, PsycInfo, PsycLit CCMED, and CAMbase [15]. We also screened the journal databases of relevant publishers, that is, gms, Karger, Kluwer, Krause and Pachernegg, Springer, Thieme, and Wiley-Interscience, to find relevant information. Finally, we searched the archive of the specialist library for CAM of Witten/Herdecke University for gray literature not listed in the above mentioned databases. The search terms were (naturopathy OR “integrative medicine” OR anthroposophical OR homeopathic) AND (clinic OR hospital). 

### 2.2. Inclusion and Exclusion Criteria

Articles were included if patients were treated in a hospital (no out-patient or day clinic treatment). To guarantee comparability SF-36 was the predefined outcome measure for patients' quality of life. To get a picture the sustainability of the effects, we decided to concentrate on the differences between “baseline” and “followup” with a follow-up duration of three months. Finally the aspect of “real world data” was covered and thus controlled clinical trials of a single drug or treatment were excluded.

All articles were fully read and their reference lists were checked for further relevant publications. To guarantee validity of the selection process, all abstracts of excluded papers were double checked. The complete search was performed between March and May 2012. The reporting of the results adhered to the MOOSE and QUOROM guidelines [16].

### 2.3. Data Extraction

Details of eligible studies were extracted and summarized using a data extraction sheet including the study indicators year, origin, institution, therapeutic approach, diseases, treatment duration, number of patients, and mental and physical scores of the SF-36 (mean and standard deviations at baseline and followup). Extracted data was cross-checked again.

### 2.4. Statistical Analysis

When a trial was found to be eligible, data of pre/post effects on the mental and physical scores of the SF-36 were converted into effect sizes and their standard deviation using an MS Excel sheet. We used the formulas

(1) $$\begin{array}{c} d=\frac{m_{1}-m_{2}}{\sqrt{\left(s_{1}^{2}+s_{2}^{2}\right)/2}},\;\text{STD}\left(d\right)=\sqrt{\frac{2\left(1-r\right)}{n}+\frac{d^{2}}{2\left(n-2\right)}} \end{array}$$

to calculate the effect size *d* between the two time points and its standard deviation STD(*d*) according to the recommendations of Dunlap et al. [17], where *m*_1_, *s*_1_ and *m*_2_, *s*_2_ denote the means and standard deviations of the pre- and post-SF-36 scores and *r* represents Pearsons correlation coefficient between them. In cases where the correlation between pre- and post-measures was not reported, we set *r* = 0.7, which according to [14] is a suitable upper bound.

To calculate overall estimates of the treatment effect we chose a random effects model according to the recommendations and algorithms given in Borenstein et al. [18] assuming that the studies were showing different treatment effects with some degree of unknown variability. Heterogeneity between trials was assessed by standard Chi-Square tests and the *I* ^2^ coefficient measuring the percentage of total variation across studies due to true heterogeneity rather than chance. Results were displayed using a forest plot.

## 3. Results

**Figure 1:**
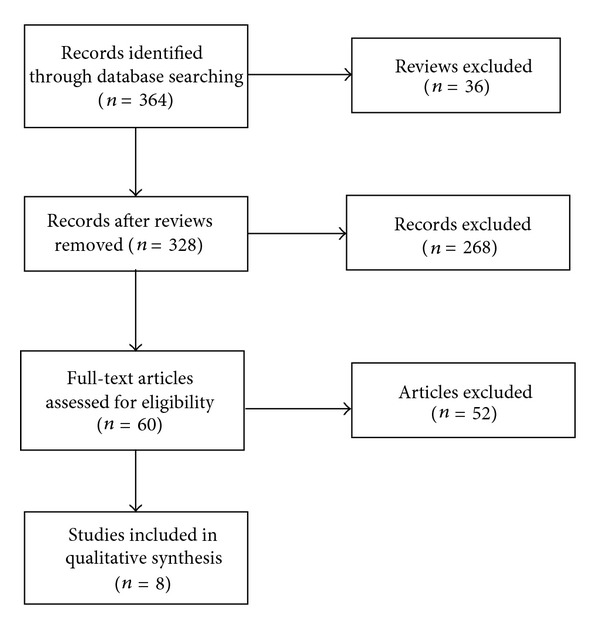
Flow chart of the inclusion process.

A total of 364 records were found, of which 36 could be identified as reviews. After screening the abstracts of the remaining 328 records, 268 records were excluded because they did not fit to the inclusion/exclusion criteria. The remaining 60 articles were assessed for eligibility and other 52 were excluded according to the inclusion/exclusion criteria after reading the full text as they provided data on out-patient treatment or did not report on SF-36 quality of life data. Thus eight articles published between 2003 and 2010 were included in the final meta-analysis. A flow chart of the inclusion process is provided in Figure 1.

Six of the eight articles described a traditional European medicine in-patient treatment strategy including the five therapeutic elements “hydrotherapy,” “phytotherapy”, “exercise therapy,” “nutrition/dietetics,” and “lifestyle modification” of classical naturopathy as originally described by Kneipp. One of the studies included “traditional Chinese medicine” as an additional therapeutic element; another one had a focus on spa therapies. The remaining two articles reported on an integrative mind body approach and on a biopsychosocial treatment strategy. Seven of the eight studies were conducted in German hospitals or hospital departments. Only one study provided data from integrative in-patient treatment from the USA.

The mean number of patients enrolled was 897 ranging from 22 to 4253. The treatment duration varied between two and three weeks. The majority of patients were treated because of diseases of the musculoskeletal system and connective tissue (ICD chapter M00–M99) including pain syndroms. The data on the 8 included articles is summarized in Table 1.

### 3.1. Meta-Analysis

Random effect meta-analysis of the eight studies revealed an overall effect size of 0.37 (95% CI: [0.28; 0.45]) in the physical score and 0.38 (95% CI: [0.30; 0.45]) in the mental scores of the SF-36. *I* ^2^ statistics indicate a high heterogeneity in the effects in both the physical and mental scores of the SF-36 (*I* ^2^ = 91.8%, *P* < 0.001, resp.; *I* ^2^ = 86.7%, *P* < 0.001).

**Figure 2:**
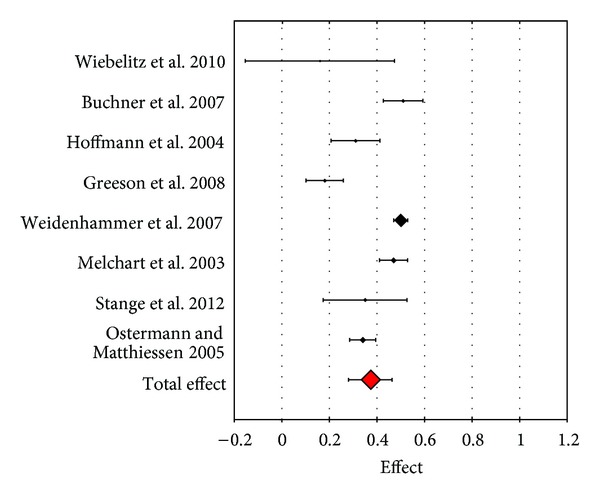
Forest plot of the effect sizes for SF-36 “physical component.”

In the physical dimension effect sizes were quite heterogenous ranging from small effects of *d* = 0.16 and *d* = 0.18 in the studies of Greeson et al. [24] and Wiebelitz et al. [25] to moderate effects of *d* = 0.50 and *d* = 0.51 in the studies of Weidenhammer et al. [22] and Buchner et al. [23] (Figure 2).

**Figure 3:**
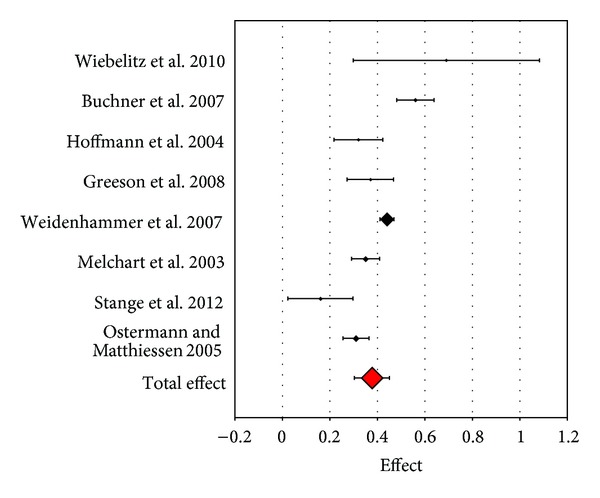
Forest plot of the effect sizes for SF-36 “mental component.”

In the mental dimension the lower bound lower bound of effect sizes is identical to the physical dimension (*d* = 0.16 in the study of Stange et al. [26]). However the upper bound sees remarkably higher effects of *d* = 0.56 in the study of Buchner et al. and *d* = 0.69 in the study of Wiebelitz et al. [25] (Figure 3).

In both dimensions the overall effect is mainly influenced by the huge cohort study of Weidenhammer et al. from 2007 [22], which included about 59% of all patients of this meta-analysis and had the second highest effect sizes in the physical score of the SF-36 (0.50 [0.48, 0.52]) and the third highest in the mental score of the SF-36 (0.44 [0.42, 0.46]). Nevertheless the results stay stable with a slightly broader confidence interval when data from Weidenhammer et al. is excluded (0.35 [0.25, 0.45], *I* ^2^ = 87.7% in the PSF-36, and 0.37 [0.28, 0.45], *I* ^2^ = 84.9% in the MSF-36).

## 4. Discussion

This is the first systematic review and meta-analysis to cover whole systems evaluations of integrative in-patient treatment. Based on the data of 7180 patients treated with integrative concepts ranging from classical naturopathy to traditional Chinese medicine we were able to calculate moderate total effect size almost three months after discharge from hospital. Quite fortunately all scientific evaluations have used standardized outcome measures and most of them included the SF-36 as a standardized measure for health related quality of life (HrQoL). Although setting parameters and patient characteristics did differ to a certain extent between the included studies, the results of this meta-analysis both from the perspective of sample size and indications and outcome measures can be regarded as a valid indicator of effectiveness for integrative in-patient treatment.

The by far most treated conditions in the 8 included studies are musculoskeletal and pain disorders [27]. It is well known from the literature that existing chronic conditions have a negative impact on HRQoL. As Langley reported, in an internet survey in Germany an estimated 24% of the adult German population reported experiencing pain in the last 30 days. Of these 13% reported severe pain. The experience of frequent severe and moderate pain has a significant deficit impact on HRQoL, both on a physical as well as a mental level [28]. This is particularly true in musculoskeletal disorders as shown by Falsarella et al. [29] who analyzed the influence of rheumatic diseases and chronic joint symptoms on the quality of life of the 2209 patients aged 60 years or over. There was a significant impact of rheumatic diseases on physical health. Furthermore joint symptoms affected self-evaluations of physical and mental health. Rheumatic diseases affected functional capacity and pain and joint symptoms relevantly affected all components of the SF-36 [29]. Thus choosing the SF-36 as outcome parameter for the present analysis is conclusive.

Without question, due to its high relevance and burden, effective multimodal interventions are needed and a moderate total effect size almost three months after discharge from hospital proves the value of this special approach especially but not exclusively in these fields of medicine. Further frequent diagnoses for integrative in-patient treatment are chronic cardiovascular, gastrointestinal or pulmonary diseases, or even oncological diseases, but currently, data to evaluate these fields of interest are lacking.

Therefore, this meta-analysis only digs a small corridor in the field of evidence. Some of the studies included in our analysis have tried to identify responders and nonresponders to integrative medicine. Although they finally did not succeed in doing so, this might still be an option if data from these studies are aggregated and reanalyzed. Apart from conducting an individual patient data meta-analysis as proposed by Vickers et al. [30], this approach may also be used to model the patient response to integrative therapies more distinctly that it can be done by a conventional meta-analysis.

However this idea is somehow limited. The fact should not be hidden that there are still several studies on whole systems evaluation of integrative in-patient treatment which have not seen the light of publication. One of the most deplorable examples in this respect is the model project Charlottenstift which aimed at integrating traditional European and traditional Chinese medicine [31]. 

Thus, this meta-analysis might be seen as an episode one of in-patient evaluation and might help to rediscover the importance of this field for patients, physicians, and stakeholders of the health care system. 

## Figures and Tables

**Table 1:** Description of the included articles.

Authors	Origin	Year	Instititution	Therapeutic approach	Diseases	Treatment duration (days)	*N*
Melchart et al. [19]	Germany	2003	TCM-Klinik, Kötzting	Classical naturopathy, traditional chinese medicine	29.7% musculoskeletal disorders 25.7% neurological disorders	N.A.	803

Hoffmann et al. [20]	Germany	2004	Knappschafts-KH, Essen	Classical naturopathy, mind body therapies	42.1% musculoskeletal disorders 17. 1% pain and migraine	14.7 ± 4.2	212

Ostermann and Matthiessen[21]	Germany	2005	Klinik Blankenstein, Hattingen	Classical naturopathy	62.7% musculoskeletal disorders 17.1% diseases of the circulatory system	21.8 ± 4.8	894

Weidenhammer et al. [22]	Germany	2007	Klinikverbund, München	Classical naturopathy, spa therapies	36.8% psychovegetative exhaustion 19.5% chronic back pain	N.A.	4253

Buchner et al. [23]	Germany	2007	Orthopädische Chirurgie, Heidelberg	Biopsychosocial therapies	100% chronic low-back pain	21	405

Greeson et al. [24]	USA	2008	Jefferson Center, Philadelphia	Integrative medicine, mind body therapies	11.8% fatigue 9.7% myalgia	N.A.	370

Wiebelitz et al. [25]	Germany	2010	Klinik Blankenstein, Hattingen	Classical naturopathy	100% chronic-low back pain	15	22

Stange et al. [26]	Germany	2012	Immanuel KH, Berlin	Classical naturopathy	41.6% low back pain 30.8% cervicobrachial syndrome	17	221
